# Impaired Shifting of Visuospatial Attention in Alzheimer’s disease as Shown by the Covert Orienting Paradigm: Implications for Visual Construction Disability

**DOI:** 10.3233/BEN-2012-110208

**Published:** 2013

**Authors:** Junichi Ishizaki, Kenichi Meguro, Nobuhiro Nara, Mari Kasai, Atsushi Yamadori

**Affiliations:** Department of Geriatric Behavioral NeurologyTohoku University Graduate School of MedicineSendaiJapan

**Keywords:** Visuospatial attention, Alzheimer’s disease, covert orienting, construction, extinction

## Abstract

*Objective:* To investigate impaired shifting of visuospatial attention in Alzheimer’s disease (AD) compared with age-matched controls.

*Method:* An attention shifting was examined in 20 AD patients and 10 age-matched normal subjects by choice reaction time (CRT) and covert orienting paradigm. Visuospatial functions tests were also performed. For covert orienting, a peripheral spatial cue method was used, with stimulus-onset (SOA) between the cue and the target time varying from 250 to 2100 ms.

*Results:* The CRT showed no difference between the AD and normal groups. However, the RTs costs plus benefits were greater in the AD than normal group for two SOA conditions independent of dementia severity. Individual profiles in the time course of cue validity revealed two AD subgroups, i.e., a normal pattern for the cue validity of time course, and an abnormal, ‘extinction-like’ pattern. The latter had a particular difficulty in performing visual construction and spatial attention.

*Conclusions:* Focusing attention was relatively intact in AD. However, shifting of visuospatial attention was impaired in AD compared with normal controls. There was a subgroup whose deficits were not only in ‘disengagement,’ but their voluntary shifting of attention was affected. These subgroups may show clinically severe visuospatial symptoms in more advanced stage.

## 1. Introduction

Posner and Petersen [32,33] proposed a model which can separate subcomponents of attention, such as orienting, target detection, and alerting. Orienting of attention has been studied in patients with brain damage using a test of visual selective attention using reaction time (RT) measurement developed by Posner [28]. Subjects are asked to press a response key after the appearance of a cue and target. The task requires a subject to continue fixing his or her attention while responding to the target, which appears in either right or left visual field. Before the target appears, a spatial cue is presented. Most cues indicate the correct location where the target is to appear (valid cues); however, some cues indicate the incorrect location contralateral to the target place (invalid cues). It is generally agreed that the valid cues enhance subjects' sensory processing at that location, thus resulting in benefits in the RT. On the contrary, the invalid cues are thought to result in costs in the RT because refocusing of attention from the incorrect to correct location is needed. Therefore, the effects of valid and invalid cues can be used to operationally define attention processing time.

Brain damage to the posterior parietal lobe was reported to have a negative effect on ability to disen-

ISSN 0953-4180/13/$27.50 © 2013 – IOS Press and the authors. All rights reserved gage from the focus of attention (one location) to a target (new location), located on the opposite side by the Posner's paradigm [25,30]. Although the neural background of such tasks is not fully understood, the view of Posner et al. [30] was also supported by PET (positron emission tomography). Namely, the posterior parietal lobe was activated by an attention shifting task from one location to another location in the contralateral field, with the right hemisphere being more specifically involved [3,26]. Since Alzheimer's disease (AD) is characterized by hypometabolism and pathological changes in the posterior parietal association cortices [9,38], AD patients may exhibit deficits of 'disengagement' on the covert orient task.

Previous studies of selective, visuospatial attention in AD patients by the covert orienting paradigm yielded several results, i.e., 'anomalous' performance (faster responses to the invalid trials) in a subgroup [6], a pattern in which focusing of attention was intact and 'disengagement' of attention was impaired [22], and right-left asymmetry of response in the visual field [13], etc. A few studies included functional imaging methods, e.g., PET [22] and SPECT (single photon emission computed tomography) [2], and confirmed the relationship between attention deficits and parietal dysfunction.

In the present study, we hypothesized that 1) AD patients show impaired shifting of attention compared with normal controls, and 2) there is a subgroup of AD patients of visuospatial attention which could explain heterogeneous neuropsychological findings in AD patients. Thus we investigated attention deficits shown by the Posner's paradigm in AD. For the purpose, we first confirmed the ability of selective attention in AD for simple choice RT (CRT) to the left or right visual target with no cues, because several studies reported decreased CRT in AD [24,27]. If AD patients had slower response in covert orienting tasks as the previous studies, we need to consider another factor such as the general slowing of cognitive processes in AD [21].

After CRT trials, we examined the deficits of selective, visuospatial attention in the AD patients using the covert orienting paradigm. Generally, RT measurements are more sensitive to component dysfunction of cognitive operations than clinical symptoms [30]. Thus it is important to understand the relationships between the deficits shown by information processing approach such as Posner's paradigm and clinical symptoms in patients. We thus clinically examined the visuospatial functions by neuropsychological tests, since the relationships between the performances of both methods are not well understood. This is the paradigm-linked study on visuospatial attention ability in AD patients using both covert orienting tasks and neuropsychological tests.

Table 1
Demographics of study population

| | AD group Mean(SD) | NC group Mean(SD) |
|---|---|---|
| Number | 20 | 10 |
| Sex (iM,F) | 3M,17F | 1M,9F |
| Age(years) | 79.4 (5.7) | 77.4 (3.9) |
| Education(years) | 8.6 (1.7) | 7.4 (1.3) |
| Mini-Mental State Examination | 17.2 (3.4) | 28.3 (1.9) |

AD = Alzheimer's disease, NC = normal control, M = male, F = female.

## 2. Methods

### 2.1. Subjects

Twenty AD patients (three men and seventeen women; mean age, 79.4 years) and 10 normal controls (one man and nine women; mean age, 77.4 years) participated in this study. They were diagnosed as probable AD according to the NINCDS-ADRDA [17]. None of these individuals showed sign of focal motor or sensory deterioration. Visual acuity of the patients was normal and they showed no visual hallucinations. They did not meet the criteria for possible or probable dementia with Lewy bodies (DLB) [16], nor did they show clinical or laboratory evidence of secondary dementia. Patients with a history of severe head injury, alcoholism, and psychiatric illness were excluded. The CT or the MRI (Magnetic Resonance Imaging) was used to reduce the possibility of including vascular dementia. The severity of these AD patients was mild to moderate, and the mean score on the Mini-Mental State Examination (MMSE) [5] was 17.2.

Normal control (NC) subjects were recruited from the community; the exclusion criteria were a history of alcoholism, psychiatric illness, significant cerebrovascular disease, head trauma, or other significant neurological conditions. NC subjects were selected whose ages matched those of the AD patients. Informed consent was obtained from the families of the patients and from the NC subjects.

Table 1 demonstrates the demographics of the study population.

*2.2. Apparatus and procedure*

*2.2.1. Choice reaction time (CRT)*

A red luminance was presented as the central fixation point on the black plastic board (30 × 45 cm) in the middle of visual field and remained visible throughout the trials. The target stimuli were presented at the same luminance to the left or right of fixation point on the horizontal meridian of board. All stimulus points were masked by a white translucent screen (15 × 30 cm). The subjects were seated approximately 80 cm from the board with the visual angle being approximately 8 degree to the right and left of target point on the horizontal meridian. They were directed to indicate detection of the target as soon as possible by pressing a computer key on the left or right side using the index finger of dominant hand or those of both hands. The stimuli remained on the board until they responded or an error. The presentation of stimuli was controlled by a personal computer and the RT data were recorded automatically in milliseconds.

*2.2.2. Covert orienting paradigm*

Two different methods for examining a covert shifting of attention are known. One method utilizes a peripheral cue which is shown at the location where the subsequent relevant event will probably occur, and the another method uses a central cue (symbol) pointing to the relevant location. Central cues are thought to require "effortful" processing, while peripheral cues are considered to draw attention involuntarily. The latter are not affected by memory load and are characterized as automatic. In the present study, we used the latter method, employing a luminous point as a peripheral spatial cue because of the following reasons. First, greater cognitive need for focal attention is required for the central cue condition, which is difficult for relatively severe AD patients. Second, non-language cues are considered to be more valid in the assessment of the visuospatial function. Third, Greenwood and Parasuraman [8] examined the effects of normal aging on visuospatial attention, both in the central and peripherally cued shift in the various age range groups (17–85 years). They reported that the cue validity on RT increased with age in the central cued paradigm and in the discrimination task but not the detection task in the peripheral cued tasks. We could avoid confusing the effect of normal aging and dementing disorders by using the peripheral, detection tasks.

Spatial cues were presented by two peripheral squares (2 × 2 cm) around one of the two target points located 8 degrees from the central fixation point in the left or right visual field. They consisted of a luminance presented for 100 milliseconds (ms) as a flank of one of the peripheral squares. The target for the task was the same red luminance to the fixation point which appeared at the center of either of the peripheral squares. In the covert orienting, a target stimulus was presented after 250, 850 and 2100 ms from the onset of the cue (Stimulus Onset Asynchrony, SOA). Spatial cues were either valid (correctly indicating the square where the target would subsequently appear) or invalid (indicating the square contralateral to the place where the target would subsequently appear). In each trial, there were approximate 70% valid cues and 30% invalid cues. Target locations were randomly divided between the right and left visual fields. During the session, the fixation point at the geometrical center of the board was shown.

*2.3. Procedure*

The subjects practiced first, and then performed the experiment randomized trials consisting of 30 trials for the CRT and 50 trials in each SOA condition (total 150 trials) for the covert orienting. The order of each SOA condition was counterbalanced across the subjects. Each trial was controlled by the examiner with judged whether the subjects were ready to respond.

*2.4. Neuropsychological tests*

All AD patients received several neuropsychological tests designed to assess three areas of higher visuospatial function, i.e., visual construction, form discrimination, and spatial attention. These tests included the followings.

*2.4.1. Visual construction*

1) Copying of the Double-Pentagon: This task is an item of the MMSE. We used standard scores on the Modified MMSE [37], which range from 0 to 10.
2) Copying of the Necker Cube: We scored this task from 0 to 2 (normal pattern = 2, incomplete pattern = 1, deterioration pattern = 0) [36].
3) Kohs Block-Design Test [10]: This test is scored from 0 to 131.

*2.4.2. Form discrimination*

1) Hidden Figures Test [12]: Patients were asked to identify the six figures of Luria's hidden figures

(three objects, one digit, and two words).

2) Benton Visual Form Discrimination Test [1]: Subjects were asked to select one item from among the four items in a multiple-choice paradigm. Each test consisted of a set of three figures, one of which matched one of the four items. There were three types of errors, i.e., displacement or rotation of the peripheral figure, rotation of one of the major figures, and distortion of one of the major figures.

*2.4.3. Spatial attention*

1) Line Bisection [35]: A variant of the Schenkenberg line bisection test was administered. Ten black lines varying in length from 12 to 20 cm were shown twice using the upside down alternate form.
2) Dot Counting [15]: A variant of the original test was administered. Patients were asked to count 14, 20, 27, and 39 dots on separate sheets of B4 (26 × 36 cm) paper.
3) Trail Making Test-A [34]: Subjects were asked to draw an unbroken line which would connect twenty-five numbered circles.

The MMSE was also performed to assess cognitive function and dementia severity of dementia.

## 3. Results

The median RTs for correct responses were computed for the simple Choice RT (CRT) and the covert orienting for each cue validity condition and each SOA (250 ms, 850 ms and 2100 ms). Reaction times shorter than 150 ms or longer than 2000 ms were defined as anticipatory responses or abnormally slow responses, respectively. They were not included in the analysis.

### *3.1. Accuracy*

The two groups were very high accurate in any conditions. Mean accuracy was 96.7% for the AD group and 99.3% for the control group in the CRT condition. In the covert orienting paradigm, it was 98.2% and 98.9%, respectively.

### *3.2. Choice reaction time*

The means of the median RTs in the CRT were 595 (± 65) ms and 507 (± 50) ms for the AD and the NC groups, respectively. The simple choice RT data were analyzed using t-test between the two groups. In the CRT, the responses of the RT in both groups were not significantly different ($p = 0.38$).

### *3.3. Covert orienting paradigm*

*3.3.1. Reaction time costs plus benefits*

The RT measurement of the deficits of the covert orienting from an invalid cue to the target can be calculated as the RT costs plus benefits (the RT to the target after an invalid cue minus the RT to the target after a valid cue).

*3.3.2. Severity and reaction time*

The RTs in the costs plus benefits showed a remarkable slow response in all SOAs conditions. No reduction of the cue validity in the longer SOA, was in contrast to the previous finding by Parasuraman et al. [22]. To examine whether this slowing of the covert orienting was linked to the general severity of dementia, the relationship of the cue validity and the severity of dementia (MMSE scores) was calculated. The correlation between the dementia severity and the RT costs plus benefits in the SOAs conditions were significant.

*3.3.3. Time course of the SOA and subgroups of AD*

Even if the AD patients had a difficulty in shifting to the contralateral field for the invalid condition (so called of ‘disengagement’ deficits) in the short (250 ms) SOA condition, the benefits of the valid conditions should be reduced and lost in longer (850 ms) SOA, because subjects are able to use voluntary shifts. Thus this slowing in the long SOA condition of the AD patients indicated other impairments of shifts in visuospatial attention as well as deficits of ‘disengagement.’ By analysis of individual patterns of time course of the SOAs, we found two different profiles in the AD patients. Namely, twelve of the 20 AD patients showed higher costs plus benefits for the 250 ms SOA condition and reduce the costs plus benefits in the longer conditions (850 ms and 2100 ms), which was the same pattern as individuals of the NC group. The remaining eight of the 20 AD patients showed paradoxically higher magnitudes for the longer SOA (850 ms and 2100 ms) conditions than the short SOA (250 ms) condition, contrary to the NC group.

*3.3.4. Relationships between deficits of covert orienting and neuropsychological performances*

For further analysis, the neuropsychological performances of the two AD subgroups were compared. As shown by Table 2, the two AD subgroups were not different with regard to age, educational level, and severity of dementia as assessed by the MMSE. Thus nei-

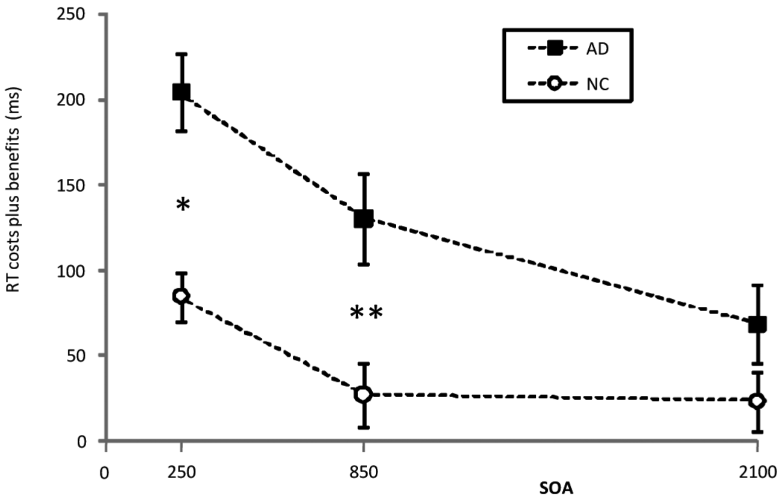

Fig. 1. Mean RT costs plus benefits for each SOA (250 ms, 850 ms and 2100 ms) condition in the AD group and the NC group. Figure 1 illustrates the mean RT costs plus benefits for the two SOA conditions. Data are presented as mean (SE) RTs for each group. The mean RT costs plus benefits of the AD and the NC groups were 184 ms and 88 ms for the 250 ms SOA condition and 137 ms and 27 ms for the 850 ms SOA condition, and 71 ms and 23 ms for the 2100 ms SOA condition, respectively. The ANOVA with repeated measures (three times) showed that the AD group had significantly higher RT costs plus benefits than the NC group ($F = 11.10$, $p = 0.003$). Significant effects of the SOA were also found ($F = 9.69$, $p < 0.001$). However there were no significant interactions of the group by the SOA ($F = 1.02$, $p = 0.37$). AD = Alzheimer's disease, NC = normal control, SOA = stimulus onset asynchrony, ms = milliseconds. The black squares were the results of the AD group while the white circles were those of the NC group.

ther age, severity of dementia, or educational level can account for the differences of the costs plus benefits. On the neuropsychological tests, the NP-AD subgroup showed a significantly better performance on the tests of visual construction (copying of the Necker Cube and the Kohs-Block Design Test) and spatial attention (dot counting and the Trail Making Test-A), suggesting that the AP-AD subgroup had more severe impairment in abilities of visual construction and visual search than the NP-AD subgroup.

## 4. Discussion

We hypothesized that 1) AD patients show impaired shifting of attention compared with normal controls, and 2) there is a subgroup of AD patients of visuospatial attention which could explain heterogeneous neuropsychological findings in AD patients.

We found that the CRT was found to show no difference between the AD and the NC groups. However, the RT costs plus benefits were found to be significantly greater in the AD patients than in the NC group for all SOA conditions. Thus the first hypothesis was proved.

From the individual profiles in the time course of cue validity, two subgroups of AD patients were found. The subgroup showing deficits of visuospatial attention for the longer SOA condition by covert orienting task had particular difficulty on the construction and spatial attention of neuropsychological tests.

### *4.1. Covert orienting and RT pattern in time course*

Parasuraman et al. [22] found that location-based, selective attention as impaired in AD patients using a covert orienting paradigm. The AD patients and the controls showed the same cue effects for different SOA conditions. Markedly increased RT cost plus benefits were found in the only short (150 ms) SOA condition, but not in the longer SOA conditions (500 ms or more). Thus they concluded that the ability to focus attention on the target was not substantially compromised in the AD, whereas they had impairment in shifting visuospatial attention, i.e., 'disengagement.' The results of our subjects indicated the similar dissociation, although our subjects were relatively more advanced cases. The mean MMSE scores were 17.2 in our patients, while 21.3 in those of Parasuraman et al. [22].

### *4.2. Subgroups of AD patients with reference to visuospatial shifting*

We first assessed the relationship between the RT costs plus benefits and the MMSE. However, no sig-

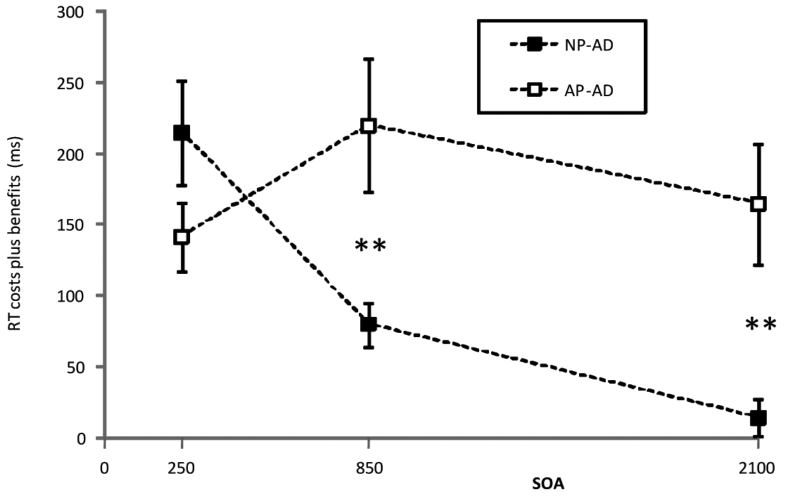

Fig. 2. Mean RT costs plus benefits for each SOA (250 ms, 850 ms and 2100 ms) condition in the two subgroups of AD. Figure 2 illustrates the costs plus benefits for each SOA in the two AD subgroups. Data are presented as mean (SE) RTs for each group. The RT costs plus benefits of the "abnormal pattern" AD subgroup (AP-AD) and those of the "normal pattern" AD subgroup (NP-AD) were significantly different by the ANOVA with repeated measures ($F = 5.86$, $p = 0.03$). A significant effect of interaction was found ($F = 11.73$, $p < 0.001$). Significant effects of the SOA were also found ($F = 10.21$, $p < 0.001$). AD = Alzheimer's disease, NP-AD = AD of normal pattern, AP-AD = AD of abnormal pattern, SOA = stimulus onset asynchrony, ms = milliseconds, The white squares were the results of the NP-AD subgroup while the black circles were those of the AP-AD subgroup.

nificant correlation was found for any SOA. Next, we analyzed individual profiles as to the time course of the cue validity and distinguished two subgroups. One had the greater costs plus benefits for the short SOA condition and the RT magnitudes were reduced for the longer SOA condition. This pattern for the time course was similar to the control group, thus we termed this AD subgroup "normal pattern (NP)"-AD subgroup. On the contrary, the other had the greater costs plus benefits for the longer SOA conditions. We termed this group "abnormal pattern (AP)"-AD subgroup.

Muller and Rabbit [20] showed that whether leading cues are peripheral or central, they involve the same, voluntary mechanism to sustain attention for periods of time longer than 400 ms. They discussed that spatial orienting could be achieved by two separate mechanism (the reflexive and the voluntary) which could be active simultaneously. Taking those facts into account, the increase of costs plus benefits in the long SOA condition in our results, the AP-AD subgroup could be affected not only by the reflexive attention but also by the voluntary attention in the covert orienting.

There is another issue to discuss regarding to the RTs slowing in the AP-AD patients for the long SOA condition. Peripheral cues have been interpreted as being associated with involuntary, automatic attention shifts as described above. The automatic process triggered by peripheral cues is most effective at short SOA [19,20, 29]. While, For the some of longer SOAs in peripheral cue paradigms, the RTs to targets appearing at uncued locations become faster than RTs to targets appearing at the cued location. This phenomenon has been called 'inhibition of return' [4,14]. Thus, it may reflect some effect of inhibitory processes for the longer SOA conditions. Inhibition of return is thought to be associated with the posterior visuospatial attention system underlying overt and covert orienting of visuospatial attention [31].

We examined the results suggesting 'inhibition of return' and found that 6 subjects in the NC group (60%) showed faster RTs on the uncued locations for the long SOA. While, 7 patients of the NP-AD group (58%) and none of the AP-AD patients (0%) showed such pattern. These results may suggest that the AP-AD patients did not show inhibitory of return. Faust and Balota [4] investigated inhibition of return in AD patients and found equivalent inhibition of return effects for AD patients, healthy older adults, and younger adults. However, facilitation and inhibition arising from the exogenous orienting of covert attention depends on the task conditions, e.g., temporal properties of spatial cues and targets [11,14]. And especially patients group of Faust and Balota [4] consisted of very mild and mild cases.

The greater effects for the invalid condition for the longer SOA conditions in the AP-AD subgroup seemed to indicate that their voluntary shifts of visuospatial at-

Table 2
Neuropsychological performances of two subgroups of AD

| Measurement | NP-AD subgroup | AP-AD subgroup | |
|---|---|---|---|
| | ($n = 12$) | ($n = 8$) | |
| Demographic | | $P$ | |
| Age(years) | 79.5 | 79.3 | n.s. |
| Education(years) | 8.2 | 9.3 | n.s |
| MMSE | 17.4 | 16.9 | n.s |
| Neuropsychological tests | | | |
| Visual construction | 9.8 | 8.5 | n.s |
| Copy of the Double Pentagon | 1.3 | 0 | $< 0.001$ |
| Copy of the Necker Cube | 23.5 | 6.6 | 0.005 |
| Kohs Block-Design Test | | | |
| From discrimination | 23.6 | 24.1 | n.s. |
| Benton Visual Form Discrimination Test | 5.4 | 4.7 | n.s. |
| Hidden Figures Test | 2.8 | 4.1 | n.s. |
| Spatial attention | 2.7 | 11 | 0.09 |
| Line Bisection(mm) | 151 | 201 | 0.03 |
| Dot Counting | | | |
| Trail Making Test-A(sec) | | | |

$p$ = level of significance between two groups by t-test.
AD = Alzheimer's disease, RT = reaction time, SOA = stimulus-onset asynchrony. NP-AD subgroup = a subgroup of AD who had a normal pattern of the RTs for time course of the SOAs conditions, AP-AD subgroup = another subgroup of AD who had an abnormal pattern of the RTs for time course of the SOAs conditions.

tention was also impaired. Although we did not find any symptom of Balint syndrome in the patients, the severe deficits of shifting attention in covert orienting may suggest certain inattention such as simultaneous agnosia. Generally, the RT measurements are more sensitive to component dysfunction of cognitive operations than clinical symptoms. Posner et al. [29] found that patients of head injury without clinical neglect showed neglect-like RT pattern in covert orienting paradigm. They called that "extinction-like" response. The pattern of the RTs in the AP-AD subgroup is better to be called a kind of 'extinction-like' or 'simultaneous agnosia-like' response.

### 4.3. *Subgroups of AD by covert orienting and neuropsychological tests*

The results of neuropsychological tests indicated that the AP-AD subgroup had severe difficulties in some tests as compared with the NP-AD subgroup. These two AD subgroups did not differ in their severity, age, and educational level. Performances on visual construction (copying of the Neker Cube and the Kohs Block Design Test) and spatial attention (dot counting and the Trail Making Test-A) were significantly lower in the AP-AD subgroup, showing that these patients had more severe impairment in visual construction and visual search functions. In AD patients, object recognition and spatial recognition were identified as different factors of deficits by a principle-factor analysis of several neuropsychological tasks assessing visuospatial abilities [7]. The authors described that the factors analysis delineated the disturbances of the two visual systems, i.e., object vision and spatial vision. Along this line, the AP-AD subgroup seemed to have more severe deficits in spatial vision but not in object vision than the NP-AD subgroup.

Our findings showed two different subgroups of AD by information processing methods and neuropsychological tests. It is known that known that some AD patients show several visuospatial disturbances, such as Balint's syndrome. Mendez et al. [18] reported that 20% (6 patients in 30) showed some of Balint's syndrome in the AD patients and those patients had poorer scores particularly on tests of spatial localization. The AP-AD patients of our study may become these type patients in more advanced stage. In fact, tree AD patients could not respond for the covert orient task, because of severe visuospatial symptoms. Further study is needed to investigate the relationship between the abilities of visuospatial attention by behavioral methods and neuropsychological tests.

## 5. Conclusions

Focusing attention was relatively intact in AD. However, shifting of visuospatial attention was impaired in

AD compared with normal controls. Previous studies have suggested that the focusing of visuospatial attention on a location was intact but that spatial shifting of attention to the contralateral field (or 'disengagement') was affected in AD [23]. We confirmed a similar pattern by CRT and covert orienting tasks in the AD patients. The RT costs plus benefits did not correlate with the dementia severity. Two AD subgroups could be divided by the RT patterns in time course of the cue validity. Although the two AD subgroups had same severity on general cognitive functions, one subgroup suggested more severe visuospatial dysfunctions on covert orienting tasks, i.e., deficits of 'inhibition of return' and decline in voluntary shifts of attention. This subgroup also showed impairments particularly in neuropsychological tests on visual construction and spatial attention.
